# TCellSI: A novel method for T cell state assessment and its applications in immune environment prediction

**DOI:** 10.1002/imt2.231

**Published:** 2024-08-26

**Authors:** Jing‐Min Yang, Nan Zhang, Tao Luo, Mei Yang, Wen‐Kang Shen, Zhen‐Lin Tan, Yun Xia, Libin Zhang, Xiaobo Zhou, Qian Lei, An‐Yuan Guo

**Affiliations:** ^1^ Hubei Bioinformatics & Molecular Imaging Key Laboratory, College of Life Science and Technology Huazhong University of Science and Technology Wuhan China; ^2^ Department of Thoracic Surgery West China Biomedical Big Data Center, West China Hospital, Sichuan University Chengdu China; ^3^ BGI Education Center University of Chinese Academy of Sciences Shenzhen China; ^4^ Center for Computational Systems Medicine, School of Biomedical Informatics The University of Texas Health Science Center at Houston Houston Texas USA

**Keywords:** T cell states, TCellSI, TCSS, immune profiling, immunotherapy

## Abstract

T cell is an indispensable component of the immune system and its multifaceted functions are shaped by the distinct T cell types and their various states. Although multiple computational models exist for predicting the abundance of diverse T cell types, tools for assessing their states to characterize their degree of resting, activation, and suppression are lacking. To address this gap, a robust and nuanced scoring tool called T cell state identifier (TCellSI) leveraging Mann–Whitney *U* statistics is established. The TCellSI methodology enables the evaluation of eight distinct T cell states—Quiescence, Regulating, Proliferation, Helper, Cytotoxicity, Progenitor exhaustion, Terminal exhaustion, and Senescence—from transcriptome data, providing T cell state scores (TCSS) for samples through specific marker gene sets and a compiled reference spectrum. Validated against sizeable pseudo‐bulk and actual bulk RNA‐seq data across a range of T cell types, TCellSI not only accurately characterizes T cell states but also surpasses existing well‐discovered signatures in reflecting the nature of T cells. Significantly, the tool demonstrates predictive value in the immune environment, correlating T cell states with patient prognosis and responses to immunotherapy. For better utilization, the TCellSI is readily accessible through user‐friendly R package and web server (https://guolab.wchscu.cn/TCellSI/). By offering insights into personalized cancer therapies, TCellSI has the potential to improve treatment outcomes and efficacy.

## Highlights

- T cell state identifier (TCellSI) is a novel and accurate scoring tool that assesses eight T cell states from transcriptome data.
- TCellSI demonstrates predictive value in the immune environment, correlating T cell states with patient prognosis and responses to immunotherapy.
- TCellSI is accessible through a user‐friendly R package and an online web server.

## Graphical abstract

TCellSI is a novel method to evaluate T cell states via transcriptome data, using specific marker gene sets and a compiled reference spectrum. TCellSI calculates T cell state scores for eight states: quiescence, regulating, proliferation, helper, cytotoxicity, progenitor exhaustion, terminal exhaustion, and senescence, offering valuable insights into the immune environment.

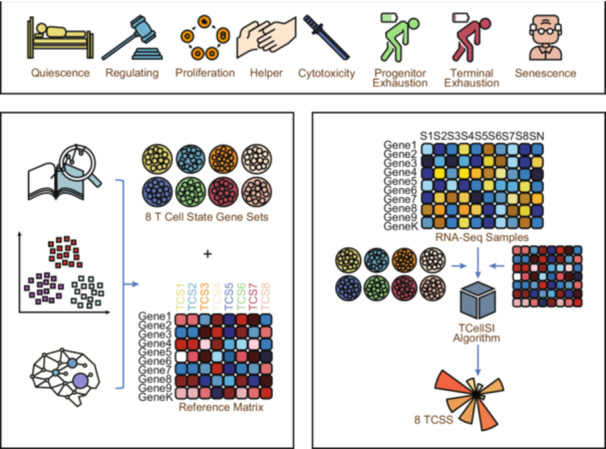

## INTRODUCTION

T cells are crucial immune cells that are essential for the immune response [1]. Recent studies have shown that T lymphocytes are incredibly diverse. They vary in their origins, differentiation trajectories, and functions, including effector, cytotoxic, and suppressive roles [2, 3]. The remarkable diversity observed among T cells is mirrored by the multitude of unique states in which they exist [4]. Many markers are shared among different T cell subtypes, suggesting that T cell states form a continuum rather than the distinct categories previously thought [5]. T cells of the same type can show varying levels of resting, activation, and suppression states. These variations result from several factors, including the local microenvironment, exposure to antigens, and signals activating the T cells [6]. Therefore, it is crucial to develop a thorough tool for assessing T cell states that accurately represent the immune environment, distinct from the subpopulations of T cells.

Targeting different states of T cells is an advanced therapeutic strategy aimed at enhancing the body's immune response to combat cancer and other diseases [7]. For instance, it has been reported that proliferative exhausted CD8 T cells enhance prolonged antitumor effects in human head and neck squamous cell carcinoma [8]. Additionally, targeting the senescent T cell immune system has been suggested as a potential strategy to prevent acute brain injuries and chronic neurodegeneration [9]. Exploring the complexities of T cell states can help us better understand how the immune system interacts with diseases, providing important clues to enhance the efficacy of immunotherapy [10]. Huge transcriptomic data including bulk RNA sequencing (RNA‐seq) and single‐cell RNA‐seq (scRNA‐seq) data offer an opportunity to identify T cell states and T cell subtypes through gene expression profiling. Tools like ImmuCellAI (immune cell abundance identifier) [11], CIBERSORTx [12], and xCell [13] allow for quantifying T cell subtypes using transcriptomic data. However, there remains a gap in methodologies specifically designed to assess the resting, activation, and suppression states of T cells in immune responses [14]. Knowing only the T cell subtypes, it remains challenging to fully understand their specific states. For example, central memory T cells (Tcm) can be either in a resting state or an activated state capable of killing [15]. Similarly, effector memory T cells (Tem) may either show advanced signs of senescence or remain relatively unaffected by senescence [16].

Therefore, there is an urgent need for an efficient method to quantify various states of T cells. In the current study, a robust and nuanced scoring tool called T cell state identifier (TCellSI) relying on the Mann–Whitney *U* statistics [17] is established. The TCellSI methodology assesses eight distinct T cell states—Quiescence, Regulating, Proliferation, Helper, Cytotoxicity, Progenitor exhaustion, Terminal exhaustion, and Senescence—and provides T cell state scores (TCSS) for samples. Validated using substantial pseudo‐bulk and actual bulk RNA‐seq data across various T cell types, TCellSI effectively identifies T cell states and outperforms established signatures in capturing the true characteristics of T cells. Importantly, this tool shows potential in oncology by linking T cell states to patient outcomes and responses to immunotherapy. TCellSI offers insights into tailored cancer therapies, potentially improving treatment results and effectiveness. The current study encompassed large‐scale data, including 379 T cell lines from 20 data sets, 34,730 single cells, 4477 pseudo‐bulk samples, 10,535 cancer patients across 33 cancer types, 7862 normal samples across 20 tissue types, 674 samples in immunotherapy cohorts, and 884 virus‐infected noncancerous peripheral blood samples.

## RESULTS

### Overview of the TCellSI method

**Figure 1:**
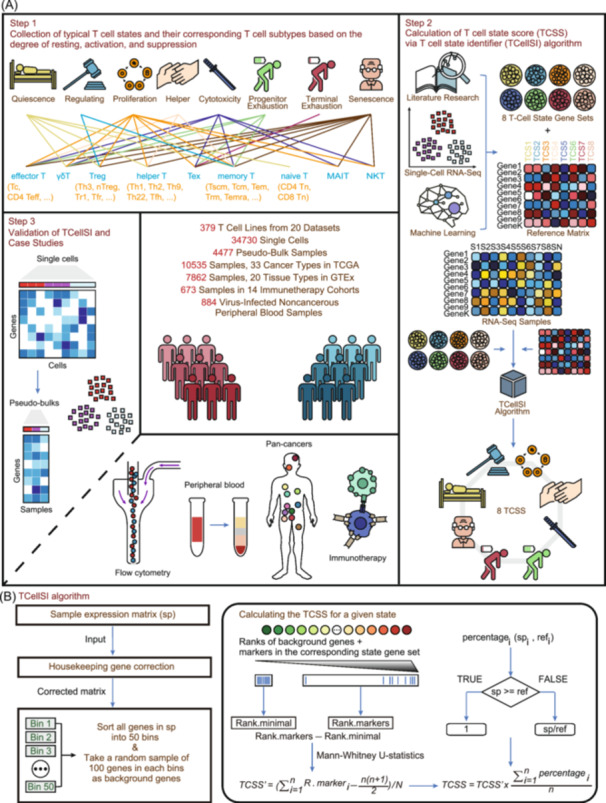
The workflow of T cell state identifier (TCellSI). (A) Step 1 displays the eight distinct T cell states and their corresponding T cell subtypes based on the degree of resting, activation, and suppression. Step 2 illustrates the algorithmic workflow of the TCellSI, detailing each phase of the process from data input to the final scoring outcome. Step 3 presents the validation of the TCellSI and includes detailed case studies that demonstrate its effectiveness in practical applications. (B) The pipeline of the TCellSI algorithm. Housekeeping gene correction ensures cross‐sample comparability. The core algorithm is founded on the principles of the Mann–Whitney *U* statistics. The reference spectrum from scRNA‐seq (single‐cell RNA sequencing) is employed to refine the scoring values. GTEx, genotype‐tissue expression; TCGA, the cancer genome atlas.

The TCellSI tool is designed to assess three main T cell states: resting, activation, and suppression. These states are further divided into eight specific states: Quiescence, Regulating, Proliferation, Helper, Cytotoxicity, Progenitor exhaustion, Terminal exhaustion, and Senescence. A specific T cell type can simultaneously exhibit high levels of different states [18, 19]. Figure 1A presents the graphic abstract of our study. Step 1 shows the eight distinct T cell states and their corresponding subtypes, categorized by their levels of resting, activation, and suppression. Step 2 outlines the TCellSI algorithmic workflow, detailing each stage from data input to the final scoring outcome. By gathering literatures, T cell single‐cell data, and employing machine learning, we derived eight marker gene sets representing different T cell states, along with a firmly established reference spectrum of these eight states. The TCellSI algorithm utilizes transcriptome data as input to evaluate eight distinct T cell states, which are presented in the form of TCSS. Step 3 demonstrates the validation of TCellSI, including detailed case studies that highlight its practical effectiveness. A brief illustration of the core algorithm of TCellSI is represented in Figure 1B, and its detailed algorithm is described in the Methods section. TCSS calculation for transcriptome data relies on the Mann–Whitney U statistics [17]. For better utilization, we developed a user‐friendly R package (https://github.com/GuoBioinfoLab/TCellSI/) and an online web server (https://guolab.wchscu.cn/TCellSI/).

### Evaluating TCellSI with simulated data generated by single‐cell RNA‐seq data

**Figure 2:**
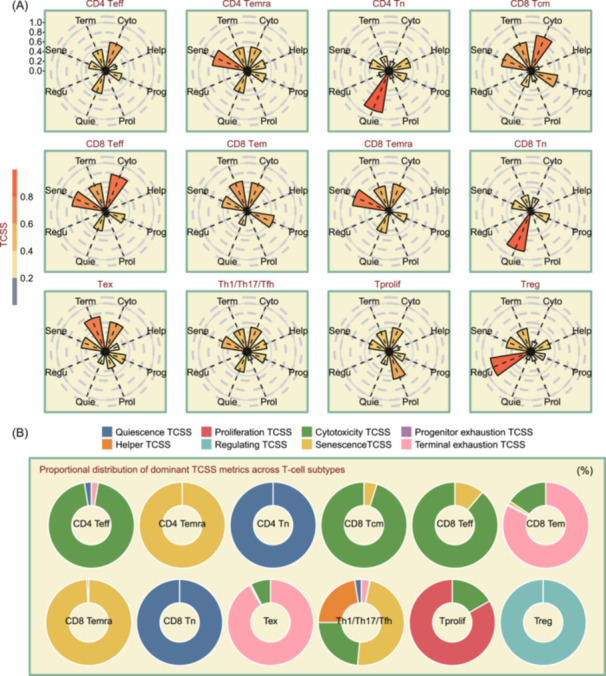
Evaluating T cell state identifier (TCellSI) with simulated data generated by scRNA‐seq data. (A) The distribution of T cell state scores (TCSS) across T cell pseudo‐bulk samples obtained from scRNA‐seq (single‐cell RNA sequencing) data. Each subplot represents the average distribution of the eight TCSS for a specific T cell subtype. (B) Proportional distribution of dominant (highest) TCSS metrics across T cell subtypes. Each doughnut chart reveals which of the eight TCSS is most prevalent within samples from a specific T cell subtype.

To validate whether TCSS can accurately represent T cell states, we utilized the TCellSI algorithm to analyze pseudo‐bulk RNA‐seq data generated by single‐cell data obtained from peripheral blood, tumor, and adjacent normal tissues of hepatocellular carcinoma (GSE98638) which include 5063 single T cells [20]. These data have 14 distinct T cell types including effector T cell (Teff), terminally differentiated effector memory T cell (Temra), naive T cell (Tn), Tcm, Tem, exhausted T cell (Tex), T helper type 1 (Th1), T helper type 17 (Th17), Tfh (follicular helper T cell), proliferating T cell (Tprolif), and regulatory T cell (Treg). We calculated TCSS across various T cell types, and each subplot of Figure 2A illustrates the average distribution of the eight TCSS for a specific T cell subtype. The results showed that Temra cells have the highest Senescence TCSS. This finding is consistent with existing theories that Temra cells usually exhibit senescence characteristics [21] such as shortened telomeres, phenotypic changes, and cell cycle arrest [22]. Meanwhile, the Temra cells have low Proliferation TCSS because of aging characteristics [23]. Tn cells exhibited the highest Quiescence TCSS among the eight metrics, and CD8 Teff and CD8 Tcm cells displayed the highest Cytotoxicity TCSS. Tex cells showed the highest scores in Terminal exhaustion TCSS, Tprolif cells exhibited the highest scores in Proliferation TCSS, and Treg cells displayed the highest scores in Regulating TCSS. Figure 2B displays the proportional distribution of dominant (highest) TCSS metrics for pseudo‐bulk RNA‐seq samples of each T cell subtype. CD4 Temra, for example, shows a Senescence TCSS percentage of 100%. This means that all CD4 Temra have the highest Senescence TCSS out of the eight TCSS measures.

**Figure 3:**
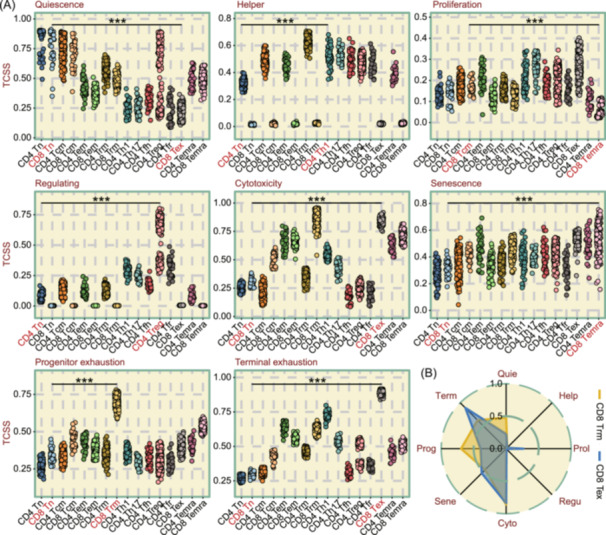
Assessing T cell state identifier (TCellSI) with T cell pseudo‐bulk samples. (A) Comparative dot plots of eight T cell state scores (TCSS) metrics across 16 T cell types using pseudo‐bulk samples derived from scRNA‐seq data. (B) Radar plot illustrating the similarities and differences in the eight TCSS between CD8 Trm (tissue‐resident memory T cell) and CD8 Tex (exhausted T cell) pseudo‐bulk samples. ***p < 0.001.

To conduct a further efficacy assessment of the TCSS, we utilized the TCellSI algorithm to analyze a broader set of single‐cell data derived from GSE108989 [24], containing 11,138 T cells including 16 T cell types. The data set encompasses Tn, Tcm, Tem, Trm (tissue‐resident memory T cell), Th1, Th17, Tfh, Treg, Tfr (follicular regulatory T cell), Tex, and Temra. We calculated TCSS using pseudo‐bulk RNA‐seq data across these various T cell types and came to the following conclusions in Figure 3A. For Quiescence TCSS, Tn cells exhibited significantly higher scores compared with other T cell types, while for Helper TCSS, CD4 T cells scored significantly higher than CD8 T cells. Temra cells scored significantly lower in the Proliferation TCSS compared with other T cell types, while Treg cells scored markedly higher in the Regulating TCSS. For Cytotoxicity TCSS, Trm, and Tex cells exhibited significantly higher scores compared with other T cell types. Indeed, CD8 Tex cells maintain substantial effector functions, expressing higher levels of granzyme, perforin, and interferon‐gamma compared with bystander cells (non‐tumor reactive) [1]. They have also been shown to proliferate more rapidly than non‐terminally Tex [25]. Regarding Senescence TCSS, Temra cells registered notably higher scores compared with other T cell types. In contrast, in terms of T cell exhaustion, Trm cells possess the highest Progenitor exhaustion TCSS, whereas Tex cells exhibit the highest Terminal exhaustion TCSS (Figure 3B). It has been demonstrated in previous studies that CD8 Trm cells have a progenitor exhausted phenotype [26, 27]. Taken together, these results suggest that TCellSI can accurately assess the state characteristics of different types of T cells.

### Validating TCellSI with bulk RNA‐seq data across multiple categories of samples

**Figure 4:**
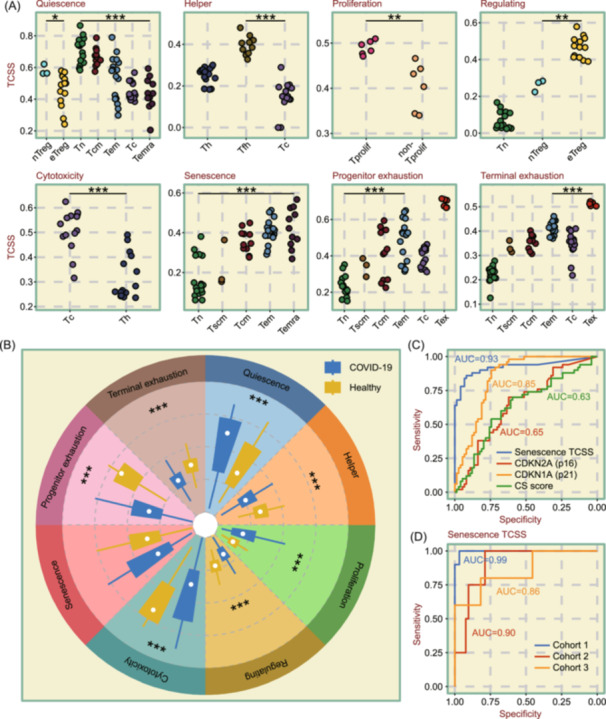
Validating T cell state identifier (TCellSI) with bulk RNA‐seq data across multiple categories of samples. (A) Dot plots comparing T cell state scores (TCSS) across bulk RNA‐seq samples of 14 different T cell types, purified from 133 samples using flow cytometry. (B) Illustrating the distribution of TCSS in peripheral blood samples from 516 COVID‐19 (Coronavirus disease 2019) patients versus 368 healthy individuals. (C) In the cohort containing 178 samples of senescent and non‐senescent T cells, the Senescence TCSS, with an AUC (area under the curve) value of 0.93, outperformed other validated markers including *p16* expression, *p21* expression, and the cellular senescence score (CS score) in predicting senescence. (D) The Senescence TCSS demonstrated superior performance, accurately predicting senescent T cells across all three cohorts with an AUC exceeding 0.85. Cohort 1 contains 105 samples, Cohort 2 contains 59 samples, and Cohort 3 contains 16 samples. *p < 0.05, **p < 0.01, ***p < 0.001.

Next, we used true bulk RNA‐seq samples of various T cell types to evaluate the performance of TCSS. We gathered 133 samples containing 14 T cell types, which were purified using flow cytometry and subsequently analyzed through bulk RNA sequencing. Figure 4A illustrates that after calculating TCSS, the Quiescence TCSS for nTreg (naive Treg) are significantly higher than those for eTreg (effector Treg), while the Regulating TCSS are significantly lower compared with eTreg. A gradual decrease in Quiescence TCSS was observed in samples consisting of Tn, Tcm, Tem, Tc (cytotoxic T cell), and Temra cell types, correlating with increasing levels of T cell differentiation. Th and Tfh cells have higher Helper TCSS, while Tc cells have higher Cytotoxicity TCSS. Th and Tfh cells have higher Helper TCSS, whereas Tc cells have higher Cytotoxicity TCSS, as is perceived. It was also confirmed that Tprolif cells exhibit markedly higher Proliferation TCSS compared with non‐Tprolif cells, and Treg cells display notably higher Regulating TCSS than Tn cells. As the degree of T cell differentiation increased, there was a gradual increase in Senescence TCSS. For both Progenitor and Terminal exhaustion TCSS, Tex cells exhibited higher scores compared with other T cell types.

To demonstrate the universality of the TCSS, we also collected and analyzed data from blood samples of 368 healthy individuals as well as 516 patients with COVID‐19 (Coronavirus disease 2019). We calculated TCSS for both groups and presented the comparisons in Figure 4B. It has been indicated that COVID‐19 patients typically experience a reduction in CD4 and CD8 T cells in peripheral blood, facilitating antiviral T cell responses [28]. Consistent with this, our findings reveal that the Quiescence TCSS was significantly higher and the Cytotoxicity TCSS markedly lower in COVID‐19 samples compared with those from healthy individuals, indicating a diminished immune status in the COVID‐19 samples.

There are a number of criteria for assessing the degree of cellular senescence, and we compared the ability of the Senescence TCSS, *p16*, and *p21* gene expression, and the CS score (cellular senescence score calculated from 1259 genes) [29] in identifying senescent cells. The test data consisted of 124 pure T cell samples (containing senescent T cells and non‐senescent T cells). We calculated the area under the curve (AUC) values of receiver operating characteristic (ROC) curves for each of the four metrics. The results demonstrated that the AUC of the Senescence TCSS reached 0.93, which exceeded that of the *p16* (AUC = 0.65), *p21* (AUC = 0.85), and CS scores (AUC = 0.63) (Figure 4C). To further validate its predictive efficacy, we compiled three additional cohorts (all containing senescent T cells and non‐senescent cells, respectively) to validate the efficacy of Senescence TCSS. In the independent validation results, the AUC values for the three cohorts were 0.99, 0.90, and 0.86, respectively (Figure 4D). These results further highlight the accuracy of the TCellSI in capturing the characteristics of T cell states.

### Impact of TCSS calculated by TCellSI on immune checkpoint blockade (ICB) therapy

**Figure 5:**
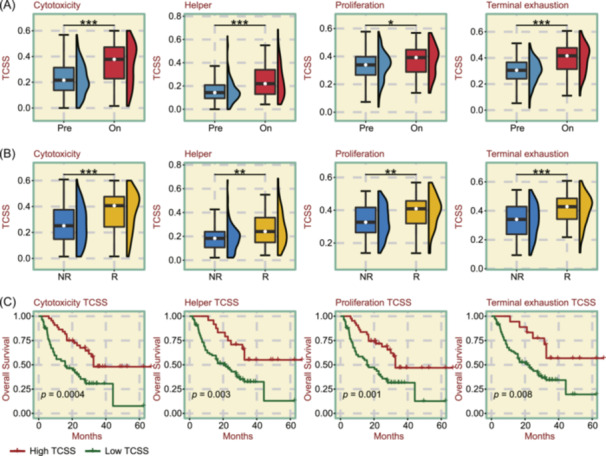
Influence of T cell state scores (TCSS) metrics on immune checkpoint blockade (ICB) therapy. (A) Significant abundance differences in the four effector TCSS metrics (Cytotoxicity, Helper, Proliferation, and Terminal exhaustion) were found between ICB pre‐treatment (Pre) and on‐treatment (On), with the On group showing higher levels in effector TCSS metrics. (B) Significant differences in the abundance of four effector TCSS metrics were observed between non‐responders (NR) and responders (R) to ICB treatment, with higher levels of effector TCSS metrics observed in the R group. (C) Survival curves demonstrated that high levels of four effector TCSS metrics were significantly correlated with prolonged overall survival (OS) in cancer patients treated with ICB. *p < 0.05, **p < 0.01, ***p < 0.001.

To examine the effects of T cell states on cancer immunotherapy outcomes, we used the TCellSI algorithm to analyze ICB treatment data, sourced from ICBatlas [30]. We collected 674 samples that received anti‐*PD‐1* therapy, anti‐*PD‐L1* therapy, anti‐*CTLA‐4* therapy, or a combination of different therapies from five cancer types of 14 data sets. We selected four effector TCSS metrics for further exploration: Cytotoxicity, Helper, Proliferation, and Terminal exhaustion. Significant increases in the abundance of the four effector TCSS metrics were observed in the ICB on‐treatment (On) phase compared with the pre‐treatment (Pre) phase of ICB therapy (Figure 5A). The results demonstrated activated Teff states after ICB treatment. Additionally, during the On phase of ICB treatment, responders (R) exhibited a more notable increase in effector TCSS compared with non‐responders (NR) (Figure 5B). Throughout the On phase of ICB treatment, patients with high effector TCSS demonstrated significantly improved overall survival (OS) compared with those with low TCSS (Figure 5C). These findings indicate that the TCSS metrics strongly correlate with immune cell infiltration during ICB therapy and may serve as a valuable marker for evaluating its effectiveness.

### Immunological profiling and prognostic implications of TCSS in pan‐cancer context

**Figure 6:**
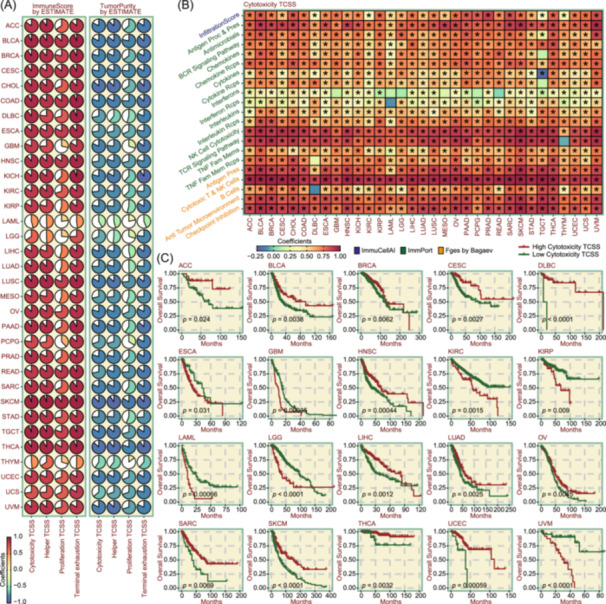
Immunological profiling and prognostic implications of T cell state scores (TCSS) in pan‐cancer context. (A) Pie charts illustrating the correlation between ImmuneScore and TumorPurity, as computed using the ESTIMATE (Estimation of STromal and Immune cells in MAlignant Tumors using Expression data) algorithm, and the four effector TCSS metrics (Cytotoxicity, Helper, Proliferation, and Terminal exhaustion) across 33 cancer types. Generally, the four effector TCSS metrics showed a positive correlation with ImmuneScore and a negative correlation with TumorPurity. (B) Heatmap depicting the correlation between three immune‐related scoring measures (InfiltrationScore calculated by ImmuCellAI (immune cell abundance identifier), antitumor pathway levels generated by the ImmPort (Immunology Database and Analysis Portal), and Fges (functional gene expression signatures) proposed by Bagaev) and Cytotoxicity TCSS across 33 cancer types. Overall, TCSS exhibited a positive association with these immune‐related scoring measures. (C) Survival curves showed that Cytotoxicity TCSS was significantly associated with overall survival (OS) in multiple cancers. *p < 0.05.

T cell function is associated with tumor immune evasion and can have a significant impact on therapeutic approaches and prognosis [31]. To investigate the impact of T cell states on tumor immunity, we applied the estimation of stromal and immune cells in malignant tumor tissues using expression data (ESTIMATE) algorithm [32] to evaluate the ImmuneScore (the proportion of immune cells) and TumorPurity (the proportion of tumor components) across 33 cancer types in The Cancer Genome Atlas (TCGA). By calculating the four effector TCSS for more than 9800 cancer samples and correlating them with ImmuneScore and TumorPurity, we observed a generally positive correlation with ImmuneScore and a negative correlation with TumorPurity (Figure 6A). For diversity validation, three immune‐related assessments were conducted: the InfiltrationScore calculated by ImmuCellAI, Antitumor pathway levels sourced from the ImmPort (Immunology Database and Analysis Portal) [33], and functional gene expression signatures (Fges) proposed by Bagaev et al [34]. Across a pan‐cancer spectrum, the InfiltrationScore tended to be higher in patients with elevated Cytotoxicity TCSS. The activity of most ImmPort pathways (antigen processing and presentation, antimicrobials, B cell receptor signaling pathway, chemokines and their receptors, cytokines and their receptors, interleukins and their receptors, natural killer (NK) cell cytotoxicity, T cell receptor signaling pathway, and tumor necrosis factor (TNF) family members and their receptors) is positively correlated with Cytotoxicity TCSS in pan‐cancer context. Also, the Fges including antigen processing, cytotoxic T and NK cells, B cells, antitumor microenvironment, and checkpoint inhibition are positively correlated with Cytotoxicity TCSS (Figure 6B). Furthermore, we investigated the effects of the Cytotoxicity TCSS on patient survival. Kaplan–Meier (KM) curves showed that Cytotoxicity TCSS was significantly associated with OS in multiple cancers. For ACC, BLCA, BRCA, CESC, DLBC, HNSC, LIHC, LUAD, OV, SARC, SKCM, THCA, and UCEC, Cytotoxicity TCSS have a positive impact on long‐term patient survival but showed an opposite trend for other cancer types, such as ESCA, GBM, KIRC, KIRP, LAML, LGG, and UVM (Figure 6C).

## DISCUSSION

Human T cells play a critical role in immune surveillance, detecting infections and other threats while maintaining protective immunity throughout the body. They are important targets for immunotherapy, transplantation, and autoimmunity [35]. However, the roles and states of T cells show remarkable diversity [36]. This diversity is reflected not only in their different types but also in the different states of resting, activation, and suppression that they may exhibit. For instance, Temra exhibits characteristics of senescence and is considered to represent senescent T cells [21, 22]. Tn is at the very beginning of differentiation and is thought to show the least immune cell activity [37]. Tex is believed to exhibit a high state of T cell exhaustion [25, 38]. This complexity highlights the need for advanced tools to accurately assess T cell states in the immune environment beyond simply identifying T cell subpopulations. Anne M. van der Leun et al. classified T cell states into four categories: naive‐like, predysfunctional, dysfunctional, and cytotoxic [39]. Building on this framework, we further divided these states into eight categories reflecting the stages T cells undergo from their initial state to senescence. These stages are Quiescence, Regulating, Proliferation, Helper, Cytotoxicity, Progenitor exhaustion, Terminal exhaustion, and Senescence.

TCellSI was developed to address these challenges by utilizing transcriptomic data to assess T cell states in tissue or blood using specific marker gene sets and a compiled reference spectrum, rather than simply estimating the abundance of T cell subpopulations. While existing methods can estimate the abundance of different types of T cells from transcriptomic data, they do not fully address the challenge of evaluating specific states of T cells in immune responses. TCSS, with scores ranging from 0 to 1, allows researchers to quantify eight different T cell states, including Quiescence, Regulating, Proliferation, Helper, Cytotoxicity, Progenitor exhaustion, Terminal exhaustion, and Senescence, making it a comprehensive method for predicting T cell states. By focusing on the state of T cells, TCellSI provides a more nuanced understanding of the immune responses.

After evaluating the TCSS against known cell type characteristics using pseudo‐bulk and actual bulk RNA‐seq data across various T cell types, it was confirmed that TCSS effectively identifies T cell states and outperforms established signatures in capturing the true characteristics of T cells. Additionally, TCSS can correctly represent T cell states in immunodeficiency diseases like COVID‐19 from blood samples. Tumor‐infiltrating T cells in various states serve as prognostic factors and predictors of therapeutic efficacy [40]. The effector TCSS metrics showed significant differences between patients based on their responses to ICB therapy, indicating that TCSS could serve as a valuable marker for evaluating the therapy's effectiveness. Additionally, Cytotoxicity TCSS was strongly linked to immune infiltration, antitumor pathway activity, antitumor functional characteristics, and prognosis across a pan‐cancer spectrum, underscoring its important role in cancer immunology research.

Several limitations in our study need to be addressed. First, the division of T cell states is not particularly detailed. The classification of T cell states needs to be studied in more detail. Second, TCSS is typically calculated using bulk RNA‐seq data. When applied to scRNA‐seq data, it is uncertain if the zero‐value issue inherent in such data will affect the calculation results and accuracy.

In summary, TCellSI provides a comprehensive framework for assessing T cell states, offering insights into the immune environment, immune deficiencies, and therapeutic effectiveness. It holds significant potential in improving immunotherapy outcomes by enabling tailored cancer treatments and advancing our understanding of T cell dynamics in various diseases.

## CONCLUSIONS

TCellSI is a powerful and innovative tool designed to assess eight distinct T cell states using transcriptome data. It has been validated against sizeable data sets across various T cell types, demonstrating its accuracy in characterizing T cell states. This tool offers significant insights into the immune environment, correlating T cell states with patient prognosis and responses to immunotherapy, thereby enhancing personalized cancer treatment strategies. TCellSI is accessible through user‐friendly R package and web server, making it a valuable resource for advancing immunological research and clinical applications.

## METHODS

### The preparation of specific marker gene sets and reference spectrum of eight T cell states

First, we established eight specific marker gene sets for each T cell state by integrating their marker genes from extensive literatures (Table S1). For the Proliferation state, which is characterized by excessive marker genes related to cytokinesis, we employed the random forest recursive feature elimination algorithm (RF‐RFE) [41, 42] to screen for important T cell proliferation‐related genes using the single‐cell Smart‐seq2 data set GSE140228 [43]. This data set comprises 119 proliferating T cells and 6773 non‐proliferating T cells. Subsequently, we identified the nine most crucial T cell proliferation‐related genes as well as T cell markers as the specific marker gene set for this state (Figure S1). The number of genes contained in the specific marker gene set of the final eight T cell states ranges from 5 to 38.

We chose to compile the TCellSI reference spectrum from T cells in the single‐cell Smart‐seq2 data obtained from the GSE99254 [44] in gene expression omnibus (GEO, http://www.ncbi.nlm.nih.gov/geo/). First, we extracted seven major classes of T cells that could represent different T cell states, including Tn, Th (Th1/Th17/Tfh), Tprolif, Treg, Tc, Terma, Tex, and finally retained a total of 11,756 single cells, which are demonstrated by Uniform Manifold Approximation and Projection (UMAP) (Figure S2A). We processed them using SAVER (single‐cell analysis via expression recovery) [45] to correct the drop‐out issue. To identify the two states of T cell exhaustion: Progenitor exhaustion and Terminal exhaustion, we performed pseudo‐time analysis by using monocle [46]. By employing this approach, we delineated the differentiation trajectories of Tex cells (Figure S2B). Differential genes identified by the pseudo‐time analysis were intersected with specific marker gene sets of the Progenitor exhaustion and Terminal exhaustion states, ultimately obtaining four genes, including *GZMB*, *HAVCR2*, *IL7R*, and *TCF7*, with distinct distributions along the trajectory (Figure S2C). Combining the distribution of these four genes, we identified Tex cells in Progenitor exhaustion and Terminal exhaustion states (Figure S2D). The eight T cell types used to establish the reference spectrum predominantly exhibit specific corresponding cell states. Tn corresponds to Quiescence, Treg corresponds to Regulating, Tprolif corresponds to Proliferation, Th corresponds to Helper, Tc corresponds to Cytotoxicity, progenitor Tex corresponds to Progenitor exhaustion, terminal Tex corresponds to Terminal exhaustion, and Temra corresponds to Senescence. Finally, the scRNA‐seq data expression profiles of the eight T cells were averaged to form the final TCellSI reference spectrum.

### Detailed algorithm and workflow of TCellSI

The RNA‐seq samples used to calculate the TCSS metrics were converted to normalized expression for TPM, and these TPM values were then log2 transformed as follows:

$$\exp_{i}\;=\log_{2}(\exp_{i}+1).$$

To facilitate comparisons across different samples in a data set, the expression levels were corrected using the expression of 3686 housekeeping genes, according to the following equation:

$$\exp_{i}^{\prime }=\exp_{i}\times \frac{\sum_{i=1}^{n}{\bar{\mathrm{HK}}}_{i}/n}{{\bar{\mathrm{HK}}}_{i}},$$

where $\exp_{i}$ represents the expression of genes in the i *‐*th sample, ${\bar{\mathrm{HK}}}_{i}$ is the average expression of total housekeeping genes in the i *‐*th sample, and n denotes the number of samples.

To control the total number of ranks, we sorted all genes in the expression matrix according to gene expression and divided them into 50 bins from high to low, and then 100 genes were randomly selected as background genes in each bin (5000 genes in total). After the above steps, the new expression matrix X (gene × sample) is obtained, and X contains the expression of the background genes plus the gene set of a certain T cell state (markers). Then, we first sorted each column in X to compute the relative rank matrix. In other words, we computed the rank list of all marker genes for each sample in X (R.marker). Based on the idea of Mann–Whitney *U*‐statistics, TCSS' is robust to data set size and heterogeneity and is calculated as follows:

$${\mathrm{TCSS}}^{\prime }=\frac{(\sum_{i=1}^{n}R.{\mathrm{marker}}_{i}-n(n+1)/2)}{N},$$

where $R.marker_{i}$ denotes the ranking value of the i *‐*th marker in the set of all markers and background genes. n indicates the number of marker genes, whereas N is the sum of the number of background genes and markers.

Furthermore, to ensure that a higher TCSS value is due to generally higher expression of each marker in its state‐specific marker gene set, rather than extreme overexpression of individual markers, which may be due to detection errors or other factors, TCellSI introduces a TPM‐normalized reference spectrum, which is used in conjunction with the TCSS' to calculate the TCSS, as shown below:

$${\mathrm{percentage}}_{i}\left({\mathrm{sp}}_{i},{\mathrm{ref}}_{i}\right)=\left\{\begin{array}{ll} 1 & \left({\mathrm{sp}}_{i}\geq {\mathrm{ref}}_{i}\right)\\ \frac{{\mathrm{sp}}_{i}}{{\mathrm{ref}}_{i}} & ({\mathrm{sp}}_{i}<{\mathrm{ref}}_{i}), \end{array}\right.$$

$$\mathrm{TCSS}={\mathrm{TCSS}}^{\prime }\;*\;\frac{\sum_{i=1}^{n}{\mathrm{percentage}}_{i}}{n},$$

where $percentage_{i}$ represents the weight of the i *‐*th marker within the marker set. The $sp_{i}$ denotes the expression level of the i *‐*th marker within the marker set in the X, whereas $ref_{i}$ refers to the expression level of the same marker in the reference spectrum. The variable n indicates the total number of marker genes. With the above process, we get TCSS for a specific T cell state. It is required to go through this process for each T cell state, incorporating the eight specific marker gene sets.

The TCSS is grounded in the relative ranking of expression levels across the eight specific marker gene sets in individual samples and is robust to minor changes in data set composition.

### Data collection and processing

For validating the TCSS, we utilized pseudo‐bulk samples derived from scRNA‐seq data accessible through GSE98638 and GSE108989 from GEO, which included diverse T cell types. The data sets contained 5063 and 11,138 T cells, respectively. We adhered to the T cell annotations provided by the original studies. We employed an under‐sampling method to generate a new batch of pseudo‐bulk samples with a single T cell subtype. During the under‐sampling, 60% of cells of a T cell subtype were randomly selected and their expression values were averaged to generate new samples. This procedure was executed multiple times, which was one‐fifth of the total cell counts per type.

Data on real bulk RNA‐seq T cell samples sorted by flow cytometry are detailed in Table S2. This includes information from 133 samples across 11 different projects: GSE199324, GSE160705, GSE232436, GSE211044, GSE173377, GSE151204, GSE179832, GSE198296, GSE145503, GSE159774, and GSE186463. The T cell types involved include naive Treg (nTreg), effector Treg (eTreg), Tn, stem memory T cell (Tscm), Tcm, Tem, cytotoxic T cell (Tc), Temra, helper T cell (Th), follicular helper T cell (Tfh), proliferating T cell (Tprolif), nonproliferating T cell (non‐Tprolif), and Tex.

The raw data cohort consisting of peripheral blood samples from 516 COVID‐19 patients was sourced from nine projects, as detailed in Table S3. These projects include ERP131828, SRP274382, SRP293106, SRP305482, SRP306910, SRP314892, SRP316381, SRP325729, and SRP359999. Additionally, 368 peripheral blood samples from healthy individuals alone were collected from 18 distinct projects (Table S4), specifically: ERP016409, ERP120543, SRP045500, SRP162348, SRP173298, SRP173378, SRP175005, SRP185630, SRP214077, SRP219679, SRP241873, SRP274382, SRP281425, SRP289418, SRP341241, SRP343650, SRP344375, and SRP312015. For the RNA‐seq raw data, data quality control was carried out with FastQC. Trimmomatic was used to eliminate adapter sequences and produce high‐quality clean reads [47]. HISAT2 [48] and Samtools [49] were subsequently employed to map these clean reads to the human reference genome GRCh38. StringTie was applied to calculate the abundance of transcripts (TPM) for each sample [50].

To evaluate the predictive efficacy of the TCSS, we collected 124 T cell samples sorted by flow cytometry sourced from nine different data sets (Table S5). These data sets, which include GSE144132, GSE173377, GSE175550, GSE180532, GSE198296, GSE199324, GSE211044, GSE216026, and GSE97862, contributed a total of 50 Temra samples and 74 samples of other T cell types. Additionally, we conducted a validation of the Senescence TCSS with 177 cell samples (Table S6), encompassing a variety of cell types including Temra, Tn, Tcm, Tem, B cells, monocytes, myeloid dendritic cells (mDC), plasma‐like cells (pDC), and NK cells. These 177 samples were organized into three cohorts: “Cohort 1” with 105 samples (GSE216529 and GSE106542); “Cohort 2” with 56 samples (GSE186463); and “Cohort 3” with 16 samples (GSE198296).

Data for ICB therapy were sourced from ICBatlas [30] and ICBcomb [51], with additional access through the database of Genotypes and Phenotypes (dbGaP, http://www.ncbi.nlm.nih.gov/gap/) and the Sequence Read Archive (SRA, http://www.ncbi.nlm.nih.gov/sra/). The melanoma samples were collected from nine published patient cohorts: Abril‐Rodriguez (dbGaP: phs001919.v1.p1), Amato (SRA: SRP250849), Auslander (SRA: SRP150548), Gide (SRA: ERP105482), Hugo (SRA: SRP070710), Liu (dbGaP: phs000452.v3.p1), Riaz (SRA: SRP094781), Van‐Allen (SRA: SRP011540), and Zappasodi (SRA: SRP302761). The non‐small cell lung cancer (NSCLC) samples were derived from the Cho (SRA: SRP183455) and Jung (SRA: SRP217040) patient cohorts. The Kim cohort (SRA: ERP107734) provided the gastric cancer (GC) samples. Renal cell carcinoma (RCC) samples came from the Miao cohort (dbGaP: phs001493.v2.p1), and the Zhao cohort (SRA: SRP155030) provided samples for glioblastoma multiforme (GBM). These data are also processed from RNA‐seq raw data, and the process is the same as previously mentioned.

The RNA‐seq data for 33 cancer types were compiled from TCGA (The Cancer Genome Atlas, https://portal.gdc.cancer.gov/) [52]. Cancer types included for further analysis were as follows: ACC, BLCA, BRCA, CESC, CHOL, COAD, DLBC, ESCA, GBM, HNSC, KICH, KIRC, KIRP, LAML, LGG, LIHC, LUAD, LUSC, MESO, OV, PAAD, PCPG, PRAD, READ, SARC, SKCM, STAD, TGCT, THCA, THYM, UCEC, UCS, and UVM (Table S7). The RNA‐seq data for 20 normal tissue types were obtained from GTEx (Genotype‐Tissue Expression, https://gtexportal.org/) [53].

References to the data in this paragraph are in the Supporting Information.

### Calculation of immune‐related traits

The ImmuneScore and TumorPurity of TCGA samples are calculated using the Estimation of STromal and Immune cells in MAlignant Tumors using Expression data (ESTIMATE) [32], while the InfiltrationScore is determined by ImmuCellAI [11]. The single‐sample gene set enrichment analysis (ssGSEA) method is utilized to evaluate immunological pathways listed in ImmPort [33], which include antigen processing and presentation, antimicrobials, B cell receptor signaling pathway, chemokines and their receptors, cytokines and their receptors, interferons and their receptors, interleukins and their receptors, NK cell cytotoxicity, T cell receptor signaling pathway, and TNF family member. Additionally, the Fges proposed by Bagaev et al. [34], which includes antigen processing, cytotoxic T and NK cells, B cells, antitumor microenvironment, and checkpoint inhibition, is also calculated through ssGSEA.

### Statistical analysis

Statistical analyses were conducted using R software (https://www.r-project.org/), version 4.1.2. Visualizations were primarily created with the “ggplot2” package in R. The processing of scRNA‐seq is based on the “Seurat” R package [54]. The “randomForest” and “caret” R packages facilitated RF‐RFE analysis. In the RF model, parameter optimization is integrated with RFE, continuously adjusting RF parameters to ensure optimal performance as the least important features are iteratively removed. This optimization includes adjusting the combination of parameters: the number of trees, the number of features considered at each split, and the minimum number of samples required at each leaf node. The “pROC” R package was used to evaluate the predictive efficiency of the TCSS through ROC curves and the calculation of AUC values. The ssGSEA was performed using the “GSVA” R package. Survival curves for two TCSS stratified groups were generated using the “survminer” R package. To assess differences between the two groups, the Wilcoxon rank sum test was applied. Spearman's correlation coefficient was used to determine the correlation between two variables. A *p*‐value of 0.05 was considered to indicate statistical significance.

## AUTHOR CONTRIBUTIONS

Jing‐Min Yang and Nan Zhang contributed equally to this work. An‐Yuan Guo, Jing‐Min Yang, and Nan Zhang conceived and designed the study. Jing‐Min Yang and Nan Zhang performed data analysis. Jing‐Min Yang and Nan Zhang wrote the manuscript. Jing‐Min Yang and Nan Zhang built the R package and the web server. Jing‐Min Yang, Tao Luo, Mei Yang, Wen‐Kang Shen, and Zhen‐Lin Tan collected data sets. Nan Zhang, Qian Lei, Yun Xia, Xiaobo Zhou, and Libin Zhang revised the manuscript. An‐Yuan Guo funded and supervised the study. All authors have read the final manuscript and approved it for publication.

## CONFLICT OF INTEREST STATEMENT

The authors declare no conflict of interest.

## ETHICS STATEMENT

No animals or humans were involved in this study.

## Supporting information


**Figure S1:** The acquisition of critical T cell proliferation‐related genes.
**Figure S2:** The preparation of compiled reference spectrum.

**Table S1:** Eight specific marker gene sets for distinct T cell states.
**Table S2:** Detailed information of real bulk RNA‐seq T cell samples sorted by flow cytometry across 11 projects, including sample sources and corresponding T cell subtypes.
**Table S3:** Cohort information from peripheral blood samples of 516 COVID‐19 patients across nine projects.
**Table S4:** Cohort information from 368 healthy individuals across 18 projects.
**Table S5:** Detailed information of 124 T cell samples sorted by flow cytometry from nine projects, including sample sources and corresponding T cell subtypes for evaluating TCSS predictive efficacy.
**Table S6:** Detailed data of 177 cell samples were sorted into three cohorts for validating Senescence TCSS, including cell types and sample sources.
**Table S7:** Detailed information on the 33 cancer types in TCGA.

## Data Availability

The data and scripts used are saved in GitHub https://github.com/GuoBioinfoLab/TCellSI/. T cell line data were obtained from GEO (http://www.ncbi.nlm.nih.gov/geo/), single‐cell data were also sourced from GEO, cancer patient data were acquired from TCGA (https://portal.gdc.cancer.gov/), normal tissue data were derived from GTEx (https://gtexportal.org/), immunotherapy cohort data were sourced from dbGaP (https://www.ncbi.nlm.nih.gov/gap/) and SRA (https://www.ncbi.nlm.nih.gov/sra/), and virus‐infected noncancerous peripheral blood sample data were obtained from SRA. Supplementary materials (methods, figures, tables, scripts, graphical abstract, slides, videos, Chinese translated version, and updated materials) may be found in the online DOI or iMeta Science http://www.imeta.science/.
