# Supplementary material for: TCellSI: A novel method for T cell state assessment and its applications in immune environment prediction
Source: Imeta. 2024 Aug 26;3(5):e231. doi: 10.1002/imt2.231 (PMC11487559; doi:10.1002/imt2.231)
Supplement: Supplementary file 1 — Figure S1: The acquisition of critical T cell proliferation‐related genes. Figure S2: The preparation of compiled reference spectrum. [file IMT2-3-e231-s002.docx]

# Supporting information to

# TCellSI: A novel method for T cell state assessment and its applications in immune environment prediction

## Running title: TCellSI for assessing T cell states

Jing-Min Yang^1,2#^, Nan Zhang^1,2#^, Tao Luo^3^, Mei Yang^1^, Wen-Kang Shen^1^, Zhen-Lin Tan^1^, Yun Xia^1^, Li-Bin Zhang^1^, Xiao-Bo Zhou^4^, Qian Lei^2^, An-Yuan Guo^2*^

^1^Hubei Bioinformatics & Molecular Imaging Key Laboratory, College of Life Science and Technology, Huazhong University of Science and Technology, Wuhan 430074, China.

^2^Department of Thoracic Surgery, West China Biomedical Big Data Center, West China Hospital, Sichuan University, Chengdu 610041, China.

^3^BGI Education Center, University of Chinese Academy of Sciences, Shenzhen 518083, China.

^4^Center for Computational Systems Medicine, School of Biomedical Informatics, The University of Texas Health Science Center at Houston, Houston, TX 77030, USA.

^#^These authors contributed equally: Jing-Min Yang, Nan Zhang

*Correspondence: [guoanyuan@wchscu.cn](mailto:guoanyuan@wchscu.cn) (An-Yuan Guo)

**Supplementary Methods**

### Data collection and processing

For validating the TCSS, we utilized pseudo-bulk samples derived from scRNA-seq data accessible through GSE98638 [1] and GSE108989 [2] from GEO, which included diverse T cell types. The datasets contained 5,063 and 11,138 T cells, respectively. We adhered to the T cell annotations provided by the original studies. For down-sampling, we employed a method where 60% of the cells from each type were randomly selected and their expression values were averaged to generate new samples. This procedure was executed multiple times with one-fifth of the total cell counts per type, to produce a new batch of pseudo-bulk T cell samples.

Data on real bulk RNA-seq T cell samples sorted by flow cytometry are detailed in Table S2. This includes information from 133 samples across 11 different projects: GSE199324 [3], GSE160705 [4], GSE232436 [5], GSE211044 [6], GSE173377 [7], GSE151204 [8], GSE179832 [9], GSE198296 [10], GSE145503, GSE159774 [11], and GSE186463 [12]. The T cell types involved include naive Treg (nTreg), effector Treg (eTreg), naive T cell (Tn), stem memory T cell (Tscm), central memory T cell (Tcm), effector memory T cell (Tem), cytotoxic T cell (Tc), terminally differentiated effector memory T cell (Temra), helper T cell (Th), follicular helper T cell (Tfh), proliferating T cell (Tprolif), non-proliferating T cell (non-Tprolif), and exhausted T cell (Tex).

The raw data cohort consisting of peripheral blood samples from COVID-19 patients were sourced from nine projects, as detailed in Table S3. These projects include ERP131828 [13], SRP274382, SRP293106 [14], SRP305482 [15], SRP306910 [16], SRP314892 [17], SRP316381 [18], SRP325729 [19], and SRP359999 [20]. Additionally, 368 peripheral blood samples from healthy individuals alone were collected from 18 distinct projects (detailed information in Table S4), specifically: ERP016409, ERP120543, SRP045500 [21], SRP162348, SRP173298, SRP173378 [22], SRP175005, SRP185630 [23], SRP214077 [24], SRP219679 [25], SRP241873, SRP274382, SRP281425 [26], SRP289418, SRP341241 [27], SRP343650 [28], SRP344375 [29], SRP312015 [30]. For the RNA-seq raw data, data quality control was carried out with FastQC. Trimmomatic was used to eliminate adapter sequences and produce high-quality clean reads. HISAT2 and Samtools were subsequently employed to map these clean reads to the human reference genome GRCh38. StringTie was applied to calculate the abundance of transcripts (TPM) for each sample.

To evaluate the predictive efficacy of the TCSS, we collected 124 T cell samples sorted by flow cytometry sourced from 9 different datasets (detailed information in Table S5). These datasets, which include GSE144132 [31], GSE173377 [7], GSE175550 [32], GSE180532 [33], GSE198296 [10], GSE199324 [34], GSE211044 [6], GSE216026 [35], GSE97862 [36], contributed a total of 50 Temra samples and 74 samples of other T cell types. Additionally, we conducted a validation of the Senescence TCSS with 177 cell samples (detailed information in Table S6), encompassing a variety of cell types including Temra, Tn, Tcm, Tem, B cells, monocytes, myeloid dendritic cells (mDC), plasma-like cells (pDC), and NK cells. These 177 samples were organized into three cohorts: "Cohort 1" with 105 samples (GSE216529 [37] and GSE106542 [38]); "Cohort 2" with 59 samples (GSE186463 [39]); and "Cohort 3" with 16 samples (GSE198296 [10]).

Data for ICB therapy were sourced from ICBatlas [40] and ICBcomb [41], with additional access through the database of Genotypes and Phenotypes (dbGaP, <http://www.ncbi.nlm.nih.gov/gap/>) and the Sequence Read Archive (SRA, <http://www.ncbi.nlm.nih.gov/sra/>). The melanoma samples were collected from nine published patient cohorts: Abril-Rodriguez [42] (dbGaP: phs001919.v1.p1), Amato [43] (SRA: SRP250849), Auslander [44] (SRA: SRP150548), Gide [45] (SRA: ERP105482), Hugo [46] (SRA: SRP070710), Liu [47] (dbGaP: phs000452.v3.p1), Riaz [48] (SRA: SRP094781), Van-Allen [49] (SRA: SRP011540), and Zappasodi [50] (SRA: SRP302761). The non-small cell lung cancer (NSCLC) samples were derived from the Cho [51] (SRA: SRP183455) and Jung [52] (SRA: SRP217040) patient cohorts. The Kim [53] cohort (SRA: ERP107734) provided the gastric cancer (GC) samples. Renal cell carcinoma (RCC) samples came from the Miao [54] cohort (dbGaP: phs001493.v2.p1), and the Zhao [55] cohort (SRA: SRP155030) provided samples for glioblastoma multiforme (GBM). These data are also processed from RNA-seq raw data, and the process is the same as previously mentioned.

The RNA-seq data for 33 cancer types were compiled from TCGA (The Cancer Genome Atlas, <https://gdac.broadinstitute.org/>). Cancer types included for further analysis were as follows: ACC, BLCA, BRCA, CESC, CHOL, COAD, DLBC, ESCA, GBM, HNSC, KICH, KIRC, KIRP, LAML, LGG, LIHC, LUAD, LUSC, MESO, OV, PAAD, PCPG, PRAD, READ, SARC, SKCM, STAD, TGCT, THCA, THYM, UCEC, UCS, UVM (detailed information in Table S7). The RNA-seq data for 20 normal tissue types were obtained from GTEx (Genotype-Tissue Expression, <https://gtexportal.org/>).

## REFERENCES

1. Zheng, Chunhong, Liangtao Zheng, Jae-Kwang Yoo, Huahu Guo, Yuanyuan Zhang, Xinyi Guo, Boxi Kang, et al. 2017. “Landscape of infiltrating T cells in liver cancer revealed by single-cell sequencing.” *Cell* 169: 1342-1356. <https://doi.org/10.1016/j.cell.2017.05.035>

2. Zhang, Lei, Xin Yu, Liangtao Zheng, Yuanyuan Zhang, Yansen Li, Qiao Fang, Ranran Gao, et al. 2018. “Lineage tracking reveals dynamic relationships of T cells in colorectal cancer.” *Nature* 564: 268-272. <https://doi.org/10.1038/s41586-018-0694-x>

3. White, Brian S., Aurélien De Reyniès, Aaron M. Newman, Joshua J. Waterfall, Andrew Lamb, Florent Petitprez, Alberto Valdeolivas, et al. 2022. “Community assessment of methods to deconvolve cellular composition from bulk gene expression.” bioRxiv <https://doi.org/10.1101/2022.06.03.494221>

4. Leem, Galam, Junsik Park, Minwoo Jeon, Eui-Soon Kim, Sang Wun Kim, Yong Jae Lee, Seong Jin Choi, et al. 2020. “4-1BB co-stimulation further enhances anti-PD-1-mediated reinvigoration of exhausted CD39+ CD8 T cells from primary and metastatic sites of epithelial ovarian cancers.” *Journal for ImmunoTherapy of Cancer* 8: e001650. <https://doi.org/10.1136/jitc-2020-001650>

5. Revu, Shankar K, Wenjuan Yang, Dhivyaa Rajasundaram, Alexander Brady, Saikat Majumder, Sarah L. Gaffen, William Hawse, Z. Xia, M. J. McGeachy. 2023. “Human IL-17A protein production is controlled through a PIP5K1α-dependent translational checkpoint.” *Science Signaling* 16: eabo6555. <https://doi.org/10.1126/scisignal.abo6555>

6. Itahashi, Kota, Takuma Irie, Junichiro Yuda, Shogo Kumagai, Tokiyoshi Tanegashima, Yi-Tzu Lin, Sho Watanabe, et al. 2022. “BATF epigenetically and transcriptionally controls the activation program of regulatory T cells in human tumors.” *Science Immunology* 7: eabk0957. <https://doi.org/10.1126/sciimmunol.abk0957>

7. Kawai, Yohei, Ai Kawana-Tachikawa, Shuichi Kitayama, Tatsuki Ueda, Shoji Miki, Akira Watanabe, Shin Kaneko. 2021. “Generation of highly proliferative, rejuvenated cytotoxic T cell clones through pluripotency reprogramming for adoptive immunotherapy.” *Molecular Therapy* 29: 3027-3041. [https://doi.org/ 10.1016/j.ymthe.2021.05.016](https://doi.org/https://doi.org/10.1016/j.ymthe.2021.05.016)

8. Renavikar, Pranav S., Sushmita Sinha, Ashley A. Brate, Nicholas Borcherding, Michael P. Crawford, Scott M. Steward-Tharp, Nitin J. Karandikar. 2020. “IL-12-induced immune suppressive deficit during CD8+ T-cell differentiation.” *Frontiers in Immunology* 11: 568630. <https://doi.org/10.3389/fimmu.2020.568630>

9. Qian, Yuan, Gabriel Arellano, Igal Ifergan, Jean Lin, Caroline Snowden, Taehyeung Kim, Jane Joy Thomas, et al. 2021. “ZEB1 promotes pathogenic Th1 and Th17 cell differentiation in multiple sclerosis.” *Cell Reports* 36: 109602. [https://doi.org/ 10.1016/j.celrep.2021.109602](https://doi.org/https://doi.org/10.1016/j.celrep.2021.109602)

10. Doan Ngoc, Tra-My, Gaëlle Tilly, Richard Danger, Orianne Bonizec, Christophe Masset, Pierrick Guérif, Sarah Bruneau, et al. 2022. “Effector memory–expressing CD45RA (TEMRA) CD8+ T cells from kidney transplant recipients exhibit enhanced purinergic p2x4 receptor–dependent proinflammatory and migratory responses.” *Journal of the American Society of Nephrology* 33: 2211-2231. <https://doi.org/10.1681/ASN.2022030286>

11. Strazza, Marianne, Shoiab Bukhari, Anna S. Tocheva, Adam Mor. 2021. “PD‐1‐induced proliferating T cells exhibit a distinct transcriptional signature.” *Immunology* 164: 555-568. <https://doi.org/10.1111/imm.13388>

12. Rose, James R., Bagdeser Akdogan-Ozdilek, Andrew R. Rahmberg, Michael D. Powell, Sakeenah L. Hicks, Christopher D. Scharer, Jeremy M. Boss. 2023. “Distinct transcriptomic and epigenomic modalities underpin human memory T cell subsets and their activation potential.” *Communications Biology* 6: 363. <https://doi.org/10.1038/s42003-023-04747-9>

13. Jackson, Heather, Irene Rivero Calle, Claire Broderick, Dominic Habgood-Coote, Giselle D’Souza, Samuel Nichols, Ortensia Vito, et al. 2022. “Characterisation of the blood RNA host response underpinning severity in COVID-19 patients.” *Scientific Reports* 12: 12216. <https://doi.org/10.1038/s41598-022-15547-2>

14. McClain, Micah T., Florica J. Constantine, Ricardo Henao, Yiling Liu, Ephraim L. Tsalik, Thomas W. Burke, Julie M. Steinbrink, et al. 2021. “Dysregulated transcriptional responses to SARS-CoV-2 in the periphery.” *Nature Communications* 12: 1079. <https://doi.org/10.1038/s41467-021-21289-y>

15. Chan, Yi‐Hao, Siew‐Wai Fong, Chek‐Meng Poh, Guillaume Carissimo, Nicholas Kim‐Wah Yeo, Siti Naqiah Amrun, Yun Shan Goh, et al. 2021. “Asymptomatic COVID‐19: disease tolerance with efficient anti‐viral immunity against SARS‐CoV‐2.” *EMBO Mol Med* 13: e14045. https://doi.org/10.15252/emmm.202114045

16. Galbraith, Matthew D., Kohl T. Kinning, Kelly D. Sullivan, Paula Araya, Keith P. Smith, Ross E. Granrath, Jessica R. Shaw, et al. 2022. “Specialized interferon action in COVID-19.” *Proceedings of the National Academy of Sciences* 119: e2116730119. <https://doi.org/10.1073/pnas.2116730119>

17. Carapito, Raphael, Richard Li, Julie Helms, Christine Carapito, Sharvari Gujja, Véronique Rolli, Raony Guimaraes, et al. 2022. “Identification of driver genes for critical forms of COVID-19 in a deeply phenotyped young patient cohort.” *Science Translational Medicine* 14: eabj7521. <https://doi.org/10.1126/scitranslmed.abj7521>

18. Sfikakis, Petros P., Kleio-Maria Verrou, Giannis Ampatziadis-Michailidis, Ourania Tsitsilonis, Dimitrios Paraskevis, Efstathios Kastritis, Evi Lianidou, et al. 2021. “Blood Transcriptomes of Anti-SARS-CoV-2 Antibody-Positive Healthy Individuals Who Experienced Asymptomatic Versus Clinical Infection.” *Frontiers in Immunology* 12: <https://doi.org/10.3389/fimmu.2021.746203>

19. Hu, Zicheng, Kattria van der Ploeg, Saborni Chakraborty, Prabhu S. Arunachalam, Diego A. M. Mori, Karen B. Jacobson, Hector Bonilla, et al. 2022. “Early immune markers of clinical, virological, and immunological outcomes in patients with COVID-19: a multi-omics study.” *eLife* 11: e77943. <https://doi.org/10.7554/eLife.77943>

20. Banerjee, Ushashi, Sneha Chunchanur, Ambica R, Kithiganahalli Narayanaswamy Balaji, Amit Singh, Dipshikha Chakravortty, Nagasuma Chandra. 2023. “Systems-level profiling of early peripheral host-response landscape variations across COVID-19 severity states in an Indian cohort.” *Genes and Immunity* 24: 183-193. <https://doi.org/10.1038/s41435-023-00210-1>

21. Linsley, Peter S., Cate Speake, Elizabeth Whalen, Damien Chaussabel. 2014. “Copy number loss of the interferon gene cluster in melanomas is linked to reduced t cell infiltrate and poor patient prognosis.” *PLoS ONE* 9: e109760. <https://doi.org/10.1371/journal.pone.0109760>

22. Catapano, Marika, Marta Vergnano, Marco Romano, Satveer K. Mahil, Siew-Eng Choon, A. David Burden, Helen S. Young, et al. 2020. “IL-36 promotes systemic ifn-i responses in severe forms of psoriasis.” *Journal of Investigative Dermatology* 140: 816-826.e813. <https://doi.org/https://doi.org/10.1016/j.jid.2019.08.444>

23. Heinrich, Maxwell J., Caroline A. Purcell, Andrea J. Pruijssers, Yang Zhao, Charles. F. Spurlock, 3rd, Subramaniam Sriram, Kristen M. Ogden, et al. 2019. “Endogenous double-stranded Alu RNA elements stimulate IFN-responses in relapsing remitting multiple sclerosis.” *J Autoimmun* 100: 40-51. <https://doi.org/10.1016/j.jaut.2019.02.003>

24. Aguirre-Gamboa, Raúl, Niek De Klein, Jennifer Di Tommaso, Annique Claringbould, Monique Gp Van Der Wijst, Dylan De Vries, Harm Brugge, et al. 2020. “Deconvolution of bulk blood eQTL effects into immune cell subpopulations.” *BMC Bioinformatics* 21: <https://doi.org/10.1186/s12859-020-03576-5>

25. Pineau, Fanny, Davide Caimmi, Milena Magalhães, Enora Fremy, Abdillah Mohamed, Laurent Mely, Sylvie Leroy, et al. 2020. “Blood co-expression modules identify potential modifier genes of diabetes and lung function in cystic fibrosis.” *PLoS ONE* 15: e0231285. <https://doi.org/10.1371/journal.pone.0231285>

26. Tabone, Olivier, Raman Verma, Akul Singhania, Probir Chakravarty, William J. Branchett, Christine M. Graham, Jo Lee, et al. 2021. “Blood transcriptomics reveal the evolution and resolution of the immune response in tuberculosis.” *Journal of Experimental Medicine* 218: e20210915. <https://doi.org/10.1084/jem.20210915>

27. Cathomas, Flurin, Laura Bevilacqua, Aarthi Ramakrishnan, Hope Kronman, Sara Costi, Molly Schneider, Kenny L. Chan, et al. 2022. “Whole blood transcriptional signatures associated with rapid antidepressant response to ketamine in patients with treatment resistant depression.” *Translational Psychiatry* 12: 12. <https://doi.org/10.1038/s41398-021-01712-0>

28. Henning, Amanda N., Daniel Green, Ryan Baumann, Patrick Grandinetti, Steven L. Highfill, Huizhi Zhou, Valeria De Giorgi. 2021. “Immunomagnetic B cell isolation as a tool to study blood cell subsets and enrich B cell transcripts.” *BMC Research Notes* 14: 418. <https://doi.org/10.1186/s13104-021-05833-z>

29. Yahara, Hiroko, Souichi Yanamoto, Miho Takahashi, Yuji Hamada, Haruo Sakamoto, Takuya Asaka, Yoshimasa Kitagawa, et al. 2022. “Whole blood transcriptome profiling identifies gene expression subnetworks and a key gene characteristic of the rare type of osteomyelitis.” *Biochemistry and Biophysics Reports* 32: 101328. <https://doi.org/10.1016/j.bbrep.2022.101328>

30. Stearrett, Nathaniel, Tyson Dawson, Ali Rahnavard, Prathyusha Bachali, Matthew L. Bendall, Chen Zeng, Roberto Caricchio, et al. 2021. “Expression of human endogenous retroviruses in systemic lupus erythematosus: multiomic integration with gene expression.” *Frontiers in Immunology* 12: 661437. <https://doi.org/10.3389/fimmu.2021.661437>

31. Williams, Kristine, Germán D. Carrasquilla, Lars R. Ingerslev, Mette Y. Hochreuter, Svenja Hansson, Nicolas J. Pillon, Ida Donkin, et al. 2021. “Epigenetic rewiring of skeletal muscle enhancers after exercise training supports a role in whole-body function and human health.” *Molecular Metabolism* 53: 101290. <https://doi.org/10.1016/j.molmet.2021.101290>

32. Yao, Wei, Xiaoxin Ren, Phillip M. Galbo Jr, Scott Moerdler, Hao Wang, R Alejandro Sica, Bijan Etemad-Gilbertson, et al. 2021. “KIR3DL3-HHLA2 is a human immunosuppressive pathway and a therapeutic target.” *Science Immunology* 6: eabf9792. <https://doi.org/10.1126/sciimmunol.abf9792>

33. Ogunshola, Funsho J., Werner Smidt, Anneta F. Naidoo, Thandeka Nkosi, Thandekile Ngubane, Trevor Khaba, Omolara O. Baiyegunhi, et al. 2022. “Hypermethylation at the CXCR5 gene locus limits trafficking potential of CD8+ T cells into B-cell follicles during HIV-1 infection.” *Blood Advances* 6: 1904-1916. <https://doi.org/10.1182/bloodadvances.2021006001>

34. Brian, S. White, Reyniès Aurélien de, M. Newman Aaron, J. Waterfall Joshua, Lamb Andrew, Petitprez Florent, Valdeolivas Alberto, et al. 2022. “Community assessment of methods to deconvolve cellular composition from bulk gene expression.” *bioRxiv* https://doi.org/10.1101/2022.06.03.494221

35. Hsiao, Cheng-Chih, Hendrik J. Engelenburg, Aldo Jongejan, Jing Zhu, Baohong Zhang, Michael Mingueneau, Perry D. Moerland, Inge Huitinga, Joost Smolders, Jörg Hamann. 2023. “Osteopontin associates with brain TRM-cell transcriptome and compartmentalization in donors with and without multiple sclerosis.” *iScience* 26: 105785. https://doi.org/10.1016/j.isci.2022.105785

36. Tian, Yuan, Mariana Babor, Jerome Lane, Veronique Schulten, Veena S. Patil, Grégory Seumois, Sandy L. Rosales, et al. 2017. “Unique phenotypes and clonal expansions of human CD4 effector memory T cells re-expressing CD45RA.” *Nature Communications* 8: <https://doi.org/10.1038/s41467-017-01728-5>

37. Sun, Xiaoming, Ce Gao, Ke Zhao, Yanhui Yang, Yelizaveta Rassadkina, Jesse Fajnzylber, James Regan, Jonathan Z. Li, Mathias Lichterfeld, Xu G. Yu. 2022. “Immune-profiling of SARS-CoV-2 viremic patients reveals dysregulated innate immune responses.” *Frontiers in Immunology* 13: 984553-984553. <https://doi.org/10.3389/fimmu.2022.984553>

38. Patil, Veena S., Ariel Madrigal, Benjamin J. Schmiedel, James Clarke, Patrick O’Rourke, Aruna D. De Silva, Eva Harris, et al. 2018. “Precursors of human CD4+ cytotoxic T lymphocytes identified by single-cell transcriptome analysis.” *Science Immunology* 3: eaan8664. <https://doi.org/10.1126/sciimmunol.aan8664>

39. Rose, James R., Bagdeser Akdogan-Ozdilek, Andrew R. Rahmberg, Michael D. Powell, Sakeenah L. Hicks, Christopher D. Scharer, Jeremy M. Boss. 2023. “Distinct transcriptomic and epigenomic modalities underpin human memory T cell subsets and their activation potential.” *Communications Biology* 6: 363. <https://doi.org/10.1038/s42003-023-04747-9>

40. Yang, Mei, Ya-Ru Miao, Gui-Yan Xie, Mei Luo, Hui Hu, Hang Fai Kwok, Jian Feng, An-Yuan Guo. 2022. “ICBatlas: a comprehensive resource for depicting immune checkpoint blockade therapy characteristics from transcriptome profiles.” *Cancer Immunology Research* 10: 1398-1406. <https://doi.org/10.1158/2326-6066.Cir-22-0249>

41. Xia, Yun, Yan Gao, Ming-Yu Liu, Lei Li, Wen Pan, Ling-Zi Mao, Zhongzheng Yang, Mei Yang, An-Yuan Guo. 2024. “ICBcomb: a comprehensive expression database for immune checkpoint blockade combination therapy.” *Briefings in Bioinformatics* 25: bbad457. <https://doi.org/10.1093/bib/bbad457>

42. Abril-Rodriguez, Gabriel, Davis Y. Torrejon, Wei Liu, Jesse M. Zaretsky, Theodore S. Nowicki, Jennifer Tsoi, Cristina Puig-Saus, et al. 2020. “PAK4 inhibition improves PD-1 blockade immunotherapy.” *Nature Cancer* 1: 46-58. <https://doi.org/10.1038/s43018-019-0003-0>

43. Amato, Carol M., Jennifer D. Hintzsche, Keith Wells, Allison Applegate, Nicholas T. Gorden, Victoria M. Vorwald, Richard P. Tobin, et al. 2020. “Pre-treatment mutational and transcriptomic landscape of responding metastatic melanoma patients to anti-PD1 immunotherapy.” *Cancers* 12: 1943. <https://doi.org/10.3390/cancers12071943>

44. Auslander, Noam, Gao Zhang, Joo Sang Lee, Dennie T. Frederick, Benchun Miao, Tabea Moll, Tian Tian, et al. 2018. “Robust prediction of response to immune checkpoint blockade therapy in metastatic melanoma.” *Nature Medicine* 24: 1545-1549. <https://doi.org/10.1038/s41591-018-0157-9>

45. Gide, Tuba N., Camelia Quek, Alexander M. Menzies, Annie T. Tasker, Ping Shang, Jeff Holst, Jason Madore, et al. 2019. “Distinct immune cell populations define response to anti-PD-1 monotherapy and anti-PD-1/anti-CTLA-4 combined therapy.” *Cancer Cell* 35: 238-255. https://doi.org/10.1016/j.ccell.2019.01.003

46. Hugo, Willy, Jesse M. Zaretsky, Lu Sun, Chunying Song, Blanca Homet Moreno, Siwen Hu-Lieskovan, Beata Berent-Maoz, et al. 2017. “Genomic and transcriptomic features of response to anti-PD-1 therapy in metastatic melanoma.” *Cell* 168: 542. https://doi.org/10.1016/j.cell.2017.01.010

47. Liu, David, Bastian Schilling, Derek Liu, Antje Sucker, Elisabeth Livingstone, Livnat Jerby-Arnon, Lisa Zimmer, et al. 2019. “Integrative molecular and clinical modeling of clinical outcomes to PD1 blockade in patients with metastatic melanoma.” *Nature Medicine* 25: 1916-1927. <https://doi.org/10.1038/s41591-019-0654-5>

48. Riaz, Nadeem, Jonathan J. Havel, Vladimir Makarov, Alexis Desrichard, Walter J. Urba, Jennifer S. Sims, F. Stephen Hodi, et al. 2017. “Tumor and microenvironment evolution during immunotherapy with nivolumab.” *Cell* 171: 934-949. https://doi.org/10.1016/j.cell.2017.09.028

49. Van Allen, Eliezer M., Diana Miao, Bastian Schilling, Sachet A. Shukla, Christian Blank, Lisa Zimmer, Antje Sucker, et al. 2015. “Genomic correlates of response to CTLA-4 blockade in metastatic melanoma.” *Science* 350: 207-211. <https://doi.org/10.1126/science.aad0095>

50. Zappasodi, Roberta, Inna Serganova, Ivan J. Cohen, Masatomo Maeda, Masahiro Shindo, Yasin Senbabaoglu, McLane J. Watson, et al. 2021. “CTLA-4 blockade drives loss of Treg stability in glycolysis-low tumours.” *Nature* 591: 652-658. <https://doi.org/10.1038/s41586-021-03326-4>

51. Park, Jaeyoon, Insang You, Sangbaie Shin, Unyong Jeong. 2015. “Material approaches to stretchable strain sensors.” *ChemPhysChem* 16: 1155-1163. https://doi.org/10.1002/cphc.201402810

52. Jung, Hyunchul, Hong Sook Kim, Jeong Yeon Kim, Jong-Mu Sun, Jin Seok Ahn, Myung-Ju Ahn, Keunchil Park, Manel Esteller, Se-Hoon Lee, Jung Kyoon Choi. 2019. “DNA methylation loss promotes immune evasion of tumours with high mutation and copy number load.” *Nature Communications* 10: 4278. <https://doi.org/10.1038/s41467-019-12159-9>

53. Kim, Seung Tae, Razvan Cristescu, Adam J. Bass, Kyoung-Mee Kim, Justin I. Odegaard, Kyung Kim, Xiao Qiao Liu, et al. 2018. “Comprehensive molecular characterization of clinical responses to PD-1 inhibition in metastatic gastric cancer.” *Nature Medicine* 24: 1449-1458. <https://doi.org/10.1038/s41591-018-0101-z>

54. Miao, Diana, Claire A. Margolis, Wenhua Gao, Martin H. Voss, Wei Li, Dylan J. Martini, Craig Norton, et al. 2018. “Genomic correlates of response to immune checkpoint therapies in clear cell renal cell carcinoma.” *Science* 359: 801-806. <https://doi.org/10.1126/science.aan5951>

55. Zhao, Junfei, Andrew X. Chen, Robyn D. Gartrell, Andrew M. Silverman, Luis Aparicio, Tim Chu, Darius Bordbar, et al. 2019. “Immune and genomic correlates of response to anti-PD-1 immunotherapy in glioblastoma.” *Nature Medicine* 25: 462-469. <https://doi.org/10.1038/s41591-019-0349-y>

**Supplementary Figures**


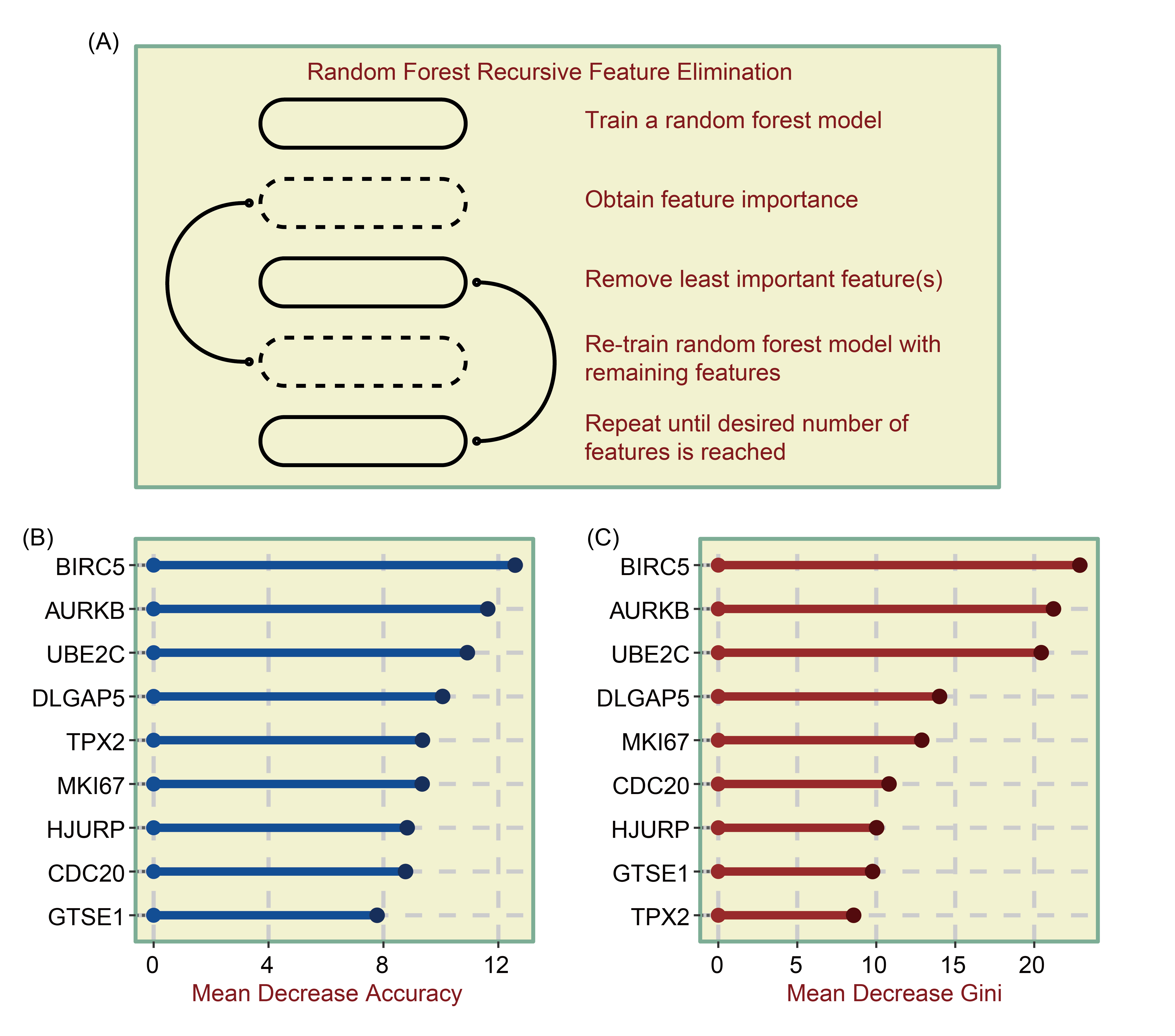


#### Figure S1 The acquisition of critical T cell proliferation-related genes. (A) The flow chart of RF-RFE. (B and C) Feature importance ranking of proliferation-related genes obtained using the RF-RFE. The variable importance by mean decrease in accuracy (B) and mean decrease gini (C) were calculated.

**
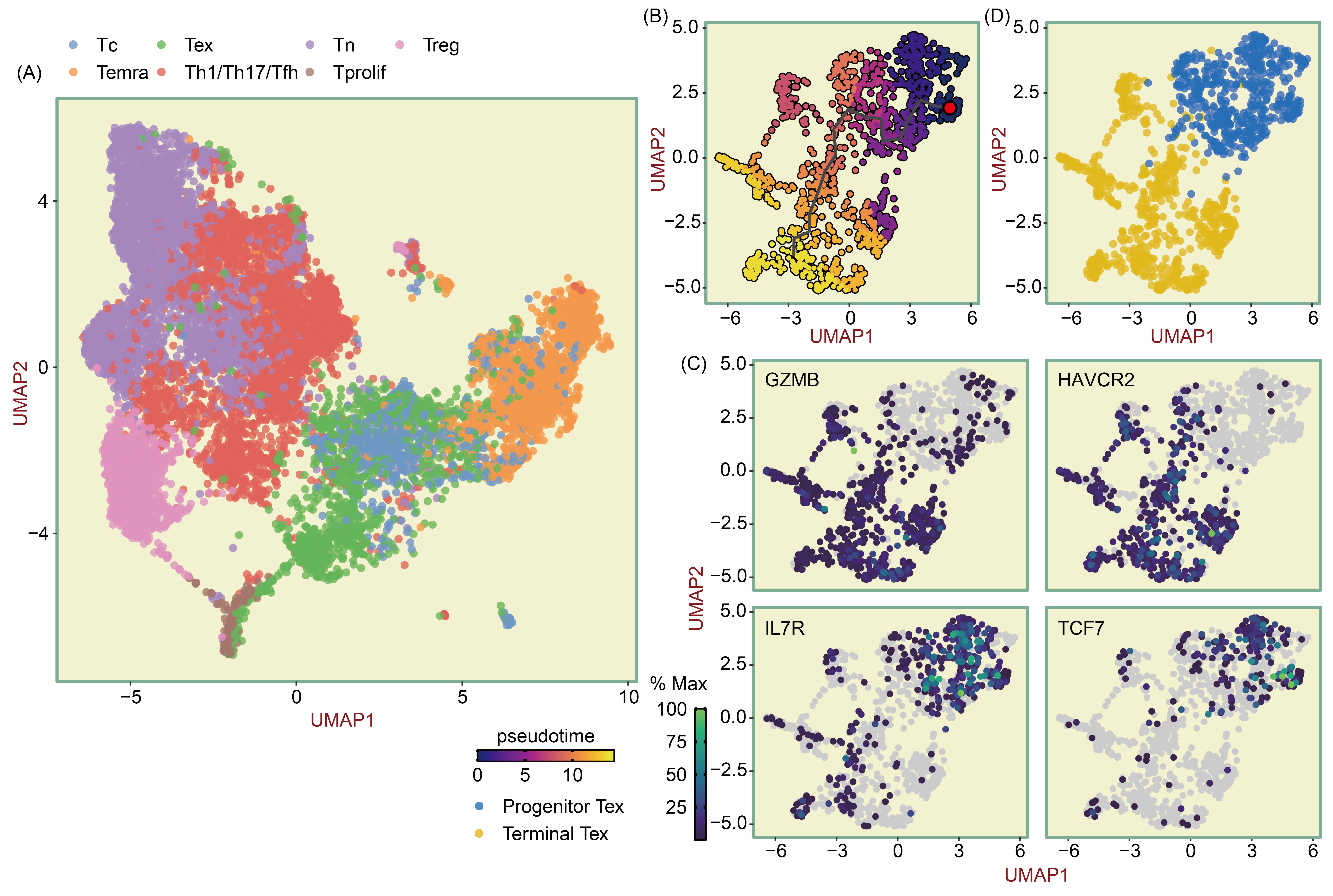
**

#### Figure S2 The preparation of compiled reference spectrum. (A) The UMAP projection of 11,756 single T cells from 14 patients. Each dot corresponds to one single cell, colored according to clusters from the database and annotations from the research of data source. (B) Single-cell trajectory analysis showing the differentiation pathway of exhausted T cells. (C) Feature plots of progenitor exhausted T cell markers (*IL7R*, *TCF7*) and terminal exhausted T cell markers (*GZMB*, *HAVCR2*), which are also differentially expressed genes of two clusters. (D) The UMAP projection of exhausted T cells according to annotations from pseudo-temporal analysis.
